# Informed choice about breast cancer prevention: randomized controlled trial of an online decision aid intervention

**DOI:** 10.1186/bcr3468

**Published:** 2013-09-03

**Authors:** Ida J Korfage, Andrea Fuhrel-Forbis, Peter A Ubel, Brian J Zikmund-Fisher, Sarah M Greene, Jennifer B McClure, Dylan M Smith, Sharon Hensley Alford, Angela Fagerlin

**Affiliations:** 1 Erasmus Medical Center, Department of Public Health, Dr. Molewaterplein 5, 3015 GE Rotterdam, the Netherlands; 2 Center for Bioethics and Social Sciences in Medicine, University of Michigan, 300 North Ingalls, Ann Arbor, MI 48106-1248, USA; 3 Division of General Internal Medicine, University of Michigan, 2800 Plymouth Road, Ann Arbor, MI 48109-2800, USA; 4 Research Center for Group Dynamics, Institute for Social Research, 426 Thompson Streeet, Ann Arbor, MI 48106-1248, USA; 5 Fuqua School of Business, Duke University, 100 Fuqua Drive, Durham, NC 27708-0120, USA; 6 Sanford School of Public Policy, Duke University, 201 Science Drive, Durham, NC 27708, USA; 7 Department of Health Behavior and Health Education, University of Michigan, 1415 Washington Hts, Ann Arbor, MI 48109-2029, USA; 8 Risk Science Center, University of Michigan, 1415 Washington Hts, Ann Arbor, MI 48109-2029, USA; 9 Group Health Research Institute, 1730 Minor Avenue, Seattle, WA 98101, USA; 10 Department of Preventive Medicine, Stony Brook University, 101 Nicolls Road, Stony Brook, NY 11794, USA; 11 Departments of Public Health Sciences & Women's Health, Henry Ford Hospital, 2799 W Grand Blvd, Detroit, MI 48202, USA; 12 VA Ann Arbor Center for Clinical Management Research, 2215 Fuller Road, Ann Arbor, MI 48105, USA; 13 Department of Psychology, University of Michigan, 530 Church Street, Ann Arbor, MI 48109-1043, USA

## Abstract

**Introduction:**

Tamoxifen and raloxifene are chemopreventive drugs that can reduce women's relative risk of primary breast cancer by 50%; however, most women eligible for these drugs have chosen not to take them. The reasons for low uptake may be related to women's knowledge or attitudes towards the drugs. We aimed to examine the impact of an online breast cancer chemoprevention decision aid (DA) on informed intentions and decisions of women at high risk of breast cancer.

**Methods:**

We conducted a randomized clinical trial, assessing the effect of a DA about breast cancer chemoprevention on informed choices about chemoprevention. Women (*n *= 585), 46- to 74-years old old, completed online baseline, post-test, and three-month follow-up questionnaires. Participants were randomly assigned to either an intervention group, a standard control group that answered questions about chemoprevention at baseline, or a three-month control group that did not answer questions about chemoprevention at baseline. The main outcome measures were whether women's intentions and decisions regarding chemoprevention drugs were informed, and whether women who viewed the DA were more likely to make informed decisions than women who did not view the DA, using a dichotomous composite variable 'informed choice' (yes/no) to classify informed decisions as those reflecting sufficient knowledge and concordance between a woman's decision and relevant attitudes.

**Results:**

Analyses showed that more intervention than standard control participants (52.7% versus 5.9%) made informed decisions at post-test, *P *<0.001. At the three-month follow-up, differences in rates of informed choice between intervention (16.9%) and both control groups (11.8% and 8.0%) were statistically non-significant, *P = *0.067.

**Conclusions:**

The DA increased informed decision making about breast cancer chemoprevention, although the impact on knowledge diminished over time. This study was not designed to determine how much knowledge decision makers must retain over time. Examining informed decisions increases understanding of the impact of DAs. A standard for defining and measuring sufficient knowledge for informed decisions is needed.

**Trial registration:**

ClinicalTrials.gov: NCT00967824

## Introduction

Tamoxifen and raloxifene are chemopreventive drugs that can reduce women's relative risk of primary breast cancer by 50% [1,2]. Tamoxifen has been available for chemoprevention for more than 10 years, and raloxifene was approved in 2006 for post-menopausal women. When the efficacy of these drugs for breast cancer prevention was established, it was initially believed they would provide a desirable option for reducing the risk of breast cancer and that these drugs might bring about a 'new era of preventive therapeutics that could revolutionize women's health'[3]. However, most women eligible for these drugs have chosen not to take them [4-8].

A number of factors relating to women's knowledge and attitudes towards these drugs appear to contribute to the low uptake. First, women are not necessarily aware of their availability - previous research found women's awareness of tamoxifen varied widely between ethnic groups, with white women most frequently reporting awareness of the drugs [9]. Second, women may not believe the drugs can truly reduce their risk of breast cancer [8,10-12]. Third, while raloxifene was originally designed to treat osteoporosis, tamoxifen was designed to prevent recurrence of breast cancer and, therefore, may have negative connotations [13]. Fourth, fear of potential side effects may inhibit positive attitudes toward taking chemoprevention drugs [5], including a perception that the potential risks (that is, increased risks of endometrial cancer, pulmonary embolism, stroke, deep vein thrombosis, cataracts, hormonal symptoms and sexual problems) outweigh the potential benefits of the drugs (that is, reduced risks of breast cancer and osteoporosis) [4,5,7,10,12,14]. Finally, many people, particularly when healthy, are opposed to taking preventive drugs on a regular basis [5,12].

Deciding whether to start chemoprevention is a preference sensitive decision, meaning there is not one superior choice [15]. To make the best decision for themselves, individuals need unbiased information [16] to consider the risks and benefits of each choice as well as their values and goals.

At present, there is no standard measure of informed choice for cancer prevention or detection behaviors. In this manuscript we adapt the concept of the Multidimensional Measure of Informed Choice (MMIC) as developed by Marteau and colleagues, which relates to the dimensions of knowledge, people's attitudes regarding the choice options and the extent to which choice reflects attitudes [17,18]. This concept was, in turn, adapted from O'Connor and O'Brien-Pallas' [19] definition of an effective decision: 'one that is based on relevant knowledge, consistent with the decision-maker's values and behaviorally implemented.' Marteau's definition states that an informed choice to engage in chemoprevention behavior occurs when a woman: 1) has sufficient relevant knowledge about chemoprevention behavior; 2) has positive attitudes toward engaging in chemoprevention; and 3) engages in chemopreventive behavior. It also occurs when a woman: 1) has sufficient relevant knowledge about chemoprevention behavior; 2) negative attitudes toward engaging in chemoprevention; and 3) does not engage in chemopreventive behavior [17]. Although decisions resulting from other combinations of these factors are traditionally considered uninformed, either because of insufficient decision-relevant knowledge or because of incongruence between attitudes and behavior, we define a third type of informed decision; specifically, women with: 1) sufficient relevant knowledge; 2) ambivalent attitudes toward chemoprevention; and 3) who are undecided or uncertain about whether to engage in chemoprevention. Chemoprevention decisions are not time-sensitive in the same way that treatment decisions are and waiting to make a choice as a result of ambivalence is quite rational, given there is little cost to delay. We characterize the choice to delay decision-making about chemoprevention due to ambivalent or neutral attitudes as informed since women may still be in the process of making a decision; their lack of a decision accurately reflects their ambivalent attitudes.

To date, we are unaware of other studies that have assessed the impact of a decision aid (DA) on women's informed decisions about breast cancer chemoprevention. Aiming to address this gap, a randomized controlled trial was conducted in 2007/2008 to test a tailored, online breast cancer chemoprevention DA. The 'Guide to Decide' DA provided tailored information (see section 2.3 for details) to women at increased risk of breast cancer about the risks and benefits of tamoxifen and raloxifene [20]. Women's knowledge, attitudes and behavioral intentions to take chemoprevention drugs were assessed immediately after viewing the DA and their uptake behavior three months later. The primary outcomes were whether women's decisions regarding chemoprevention drugs were informed and whether women who viewed the DA were more likely to make informed decisions than women who did not view the DA.

## Methods

### Participant recruitment

After obtaining Institutional Review Board approval from the University of Michigan and two health care delivery systems (Henry Ford Health System in Detroit, MI and Group Health Cooperative in Seattle, WA) and registering the trial (http://ClinicalTrials.gov; number NCT00967824), electronic medical records were used to create a list of eligible women - specifically women at high risk of developing invasive breast cancer, defined by a five-year risk of ≥1.66% on the Breast Cancer Risk Assessment Tool (BCRAT, based on the Gail model) [19-21] and without contraindications to tamoxifen or raloxifene. We sent these women invitation letters with a description of the study, the study website, and a username and password. There was no racial bias in the selection of participants. Interested women logged in and were screened for final eligibility based on self-report. Eligible women were between 40 and 74 years old, (as chemoprevention is only approved for those ages) and were post-menopausal (raloxifene is only approved for post-menopausal women [21]). Women were excluded if they reported a prior history of breast cancer or tamoxifen or raloxifene use, contraindications to tamoxifen or raloxifene, participation in the Study of Tamoxifen and Raloxifene (STAR) trial [21], terminal illness, or were currently pregnant or nursing.

### Randomization to intervention and control groups

**Figure 1:**
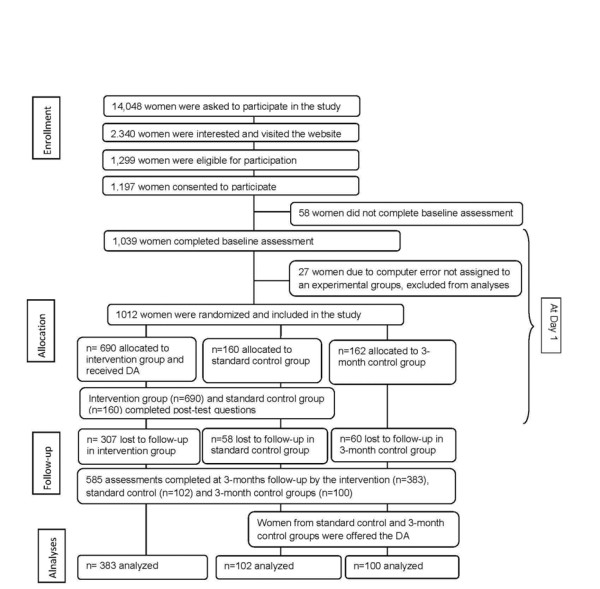
**Flow diagram of study populations and assessment**.

A total of 1,039 women gave their informed consent to participate in the study. Due to a computer error, only 1,012 of them were correctly randomized (see Figure 1, the flow diagram). This report is limited to the 585 of the 1,039 (57.8%) women who completed the three-month follow-up survey.

After completing eligibility screening and the baseline questionnaire, participants were randomly assigned to one of three groups: intervention, standard control, or three-month control as previously described by Fagerlin and colleagues [8] (Figure 1). Intervention group participants received the Guide to Decide DA (described below) immediately after randomization and then completed a post-test questionnaire. Standard control group participants did not receive the DA until they completed the three-month follow-up, but completed the same questionnaires as the intervention group. Since participants in the standard control group might seek information (that is, "Google' tamoxifen and raloxifene) after having answered questions about chemoprevention without receiving any information, we included the three-month control group. Participants in this group answered chemoprevention questions at the three-month follow-up, but neither received the DA nor answered any questions about chemoprevention at the time of enrollment, which ensured the inclusion of a control group without previous exposure to chemoprevention information. Analyses examining the immediate effect of the DA compare participants from the intervention group to the standard control group, and analyses examining the longer-term effect of the DA compare participants from the intervention group to both control groups.

### The 'Guide to Decide' decision aid intervention

The Guide to Decide DA provided general information about breast cancer, tamoxifen and raloxifene [1]. Women received tailored (personalized) information about their individual five-year risk of breast cancer according to their scores on the BCRAT [20]. Side effect rates were tailored to each woman's age and race. This information included estimates of the risks of endometrial cancer, blood clotting problems, cataracts, hormone symptoms, sexual problems, breast cancer and bone fractures. The DA was written at approximately an eighth grade reading level. Further details about the development and tailoring of the Guide to Decide DA intervention have been published previously [8].

### Measures

#### Knowledge of risks and benefits of chemoprevention

Women's gist knowledge ('the ability to identify the essential point of the information presented') of the risks and benefits of each drug was measured through questions such as, 'Please tell us whether you think taking tamoxifen (or raloxifene) will make you less likely, more likely, or have no difference in the likelihood that you would experience each of the health conditions listed below.' Participants were then given a list of two risks (increased risk of hormonal symptoms and cataracts) and two benefits (decreased risk of broken bones and breast cancer) of tamoxifen (or raloxifene) and were asked to answer the likelihood question for each risk and benefit. Each question was asked for both tamoxifen and raloxifene. The intervention and standard control groups answered these knowledge questions at post-test and three-month follow-up, while the three-month control group only answered them at three-month follow-up.

Based on other researchers' work, we assessed distributions of accurate knowledge in the data and operationalized a 'sufficient level of decision-relevant knowledge' as answering at least 50% of the items (4 of 8) correctly and 'insufficient decision-relevant knowledge' as three or fewer correctly answered items [22].

#### Attitudes toward chemopreventive drugs

Women were asked, 'How good of a choice is taking tamoxifen (or raloxifene) as a way to reduce your chance of getting breast cancer?' (1 = 'For me it is not a good choice at all,' to 5 = 'For me it is an extremely good choice'). Response options were worded to assess attitudes toward own behavior rather than attitudes toward tamoxifen in general (for example, a woman may feel chemoprevention is a good option for others but feel it is not a good choice for her personally). Responses 1 and 2 were classified as 'negative attitudes' and responses 4 and 5 as 'positive attitudes'. The midpoint response option 3 was classified as 'neutral attitudes' [22].

#### Behavioral intentions

Behavioral intentions to take chemoprevention drugs in the post-test survey were measured using 'Given what you know right now, how likely doyou think you are to take a breast cancer prevention drug in the next year?' (1 = 'not at all likely*,' *to 5 = 'extremely likely'). Responses 1 and 2 were classified as not likely to engage in chemoprevention behavior, response 3 was classified as neutral about engagement, and responses 4 and 5 were classified as likely to engage in chemoprevention behavior.

#### Actual behavior

Chemoprevention uptake behavior was measured in all three groups at follow-up by asking participants, 'Have you made a decision about whether or not to take a breast cancer prevention drug as a way to prevent breast cancer?' (1 = 'no decision yet'; 2 = 'decided to take Tamoxifen'; 3 = 'decided to take Raloxifene'; 4 = 'decided to take no drug').

### Informed or uninformed decision

A dichotomous variable was created, 'informed decision,' (yes/no) representing participants with sufficient knowledge about chemoprevention behavior (assessed at post-test and follow-up), whose attitudes were concordant with their intentions (at post-test) or decisions (at follow-up) to engage in chemoprevention behavior.

According to Fuzzy Trace Theory [23], it is possible that participants made an initial decision at post-test based on their knowledge and attitudes at that time, and then forgot details by follow-up but remembered their general decision. These participants would, therefore, make a decision at follow-up based on their initial (post-test) knowledge and feelings. Three different 'informed decision' scores were calculated to account for the various ways participants may have arrived at an informed decision. Post-test knowledge, attitudes and intentions were used for the 'post-test informed decision' score. Follow-up knowledge, attitudes and behavior were used for the 'follow-up informed decision' score. Finally, post-test knowledge and follow-up attitudes and behavior were used for the 'latent knowledge informed decision' score.

### Covariates

At baseline, age, race and ethnicity, and educational background were assessed. Educational background was condensed into three levels of education (high school or less, some college or trade school, and bachelor's degree or higher).

Participants completed standardized individual difference measures of subjective numeracy [24,25] and health, using the single item, 'How would you rate your health?*' *(1 = poor, 2 = fair, 3 = good, 4 = very *good *and 5 = excellent).

### Analyses

We calculated means, standard deviations and frequencies to describe the participants and experimental groups. Inferential statistics include t-tests, analysis of variance, and Pearson chi-square analysis (2-tailed significance).

## Results

### Participants

A total of 585 women completed the study through the three-month follow-up (see Figure 1 for details on participant flow through the intervention). Table 1 describes participants' baseline characteristics. On average, participants were 61.6 (SD = 5.2) years old, mostly white (98%) and well educated (71% had at least a bachelor's degree). BCRAT scores ranged from 1.7% to 19.1% (M = 2.67, SD = 1.37).

**Table 1:** Baseline variables for women who completed entire study

	Intervention (*n *= 383)	Standard control (*n *= 102)	Three-month control (*n *= 100)	Between-groups differences
Age, mean (SD)	61.50 (5.05)	62.10 (5.25)	61.51 (5.63)	F(2, 582) = 0.55, *P *= .58
Age, over 60	249 (65.0%)	67 (65.7%)	65 (65.0%)	X^2^(2) = 0.02, *P *= .99
Education				
High school education or less	19 (5.0%)	3 (2.9%)	7 (7.0%)	X^2^(4) = 3.69, *P *= .45
Some college or trade school	84 (22.2%)	29 (28.4%)	26 (26.0%)	
Four-year college degree or more	276 (72.8%)	70 (68.6%)	67 (67.0%)	
Race				
Hispanic	1 (0.3%)	0 (0%)	0 (0%)	X^2^(2) = 0.53, *P *= .77
White	374 (97.7%)	99 (97.1%)	99 (99.0%)	X^2^(2) = 0.96, *P *= .62
Black	2 (0.5%)	0 (0%)	0 (0%)	X^2^(2) = 1.06, *P *= .59
BCRAT score (SD)	2.67 (1.19)	2.55 (0.92)	2.82 (2.16)	F(2, 582) = 1.06, *P *= .35
Subjective Numeracy Scale	4.48 (0.94)	4.53 (0.95)	4.29 (0.99)	F(2, 582) = 1.87, *P *= .16

### Post-test knowledge, attitudes and behavioral intentions

More intervention participants (62%) than standard control participants (7%) met the criteria for 'sufficient knowledge,' Χ^2^(1) = 97.53, *P *<.001 (answering at least 4 out of 8 questions correctly) (Table 2).

**Table 2:** Effects of DA: post-test measures of knowledge, attitudes, and intentions

		Intervention (*n *= 383)	Standard control (*n *= 102)	Three-month control (*n *= 100) ^a^	Between-groups differences
**Post-test**					

Knowledge	Insufficient (≤50% correct)	146 (38.1%)	95 (93.1%)		Χ^2^(1 9 7.53, *P *<.001
	Sufficient		237 (61.9%)	7 (6.9%)	
Attitudes	Negative	284 (74.9%)	61 (61.6%)		Χ^2^(2) = 12.64, *P *= .002
	Neutral		72 (19.0%)	35 (35.4%)	
	Positive		23 (6.1%)	3 (3.0%)	
Intention for uptake	Not likely	323 (85.2%)	78 (77.2%)		Χ^2^(2) = 6.38, *P *= .041
	Neutral		40 (10.6%)	20 (19.8%)	
	Likely		16 (4.2%)	3 (3.0%)	
**Three-month follow-up**					
Knowledge	Insufficient (≤ 50% correct)	288 (75.2%)	87 (85.3%)	88 (88.0%)	Χ^2^(2) = 10.71, *P *= .005
	Sufficient		95 (24.8%)	15 (14.7%)	12 (12.0%)
Attitudes	Negative	280 (75.1%)	68 (69.4%)	59 (62.8%)	X^2^(4) = 7.84, *P *= .098
	Neutral		83 (22.3%)	24 (24.5%)	30 (31.9%)
	Positive		10 (2.7%)	6 (6.1%)	5 (5.3%)
Uptake at follow-up	No decision	171 (44.8%)	67 (65.7%)	70 (70.0%)	Χ^2^(4) = 29.06, *P *<.001
	Take tamoxifen		0	0	0
	Take raloxifene**^a^**		2 (0.5%)	0	0
	Decided no drug		209 (54.7%)	35 (34.3%)	30 (30.0%)

**^a ^**Three-month control group did not complete measures at post-test.

When asked 'How good of a choice is taking a breast cancer prevention drug as a way to reduce your chance of getting breast cancer?', 75% of the intervention group and 62% of the standard control group indicated negative attitudes toward taking chemoprevention themselves. Intervention participants reported 19% neutral attitudes and 6% positive attitudes, and standard control participants reported 35% neutral attitudes and 3% positive attitudes (Table 2). Distribution of attitudes varied significantly between the intervention and standard control groups, (Χ^2^(2) = 12.64, *P *= .002).

Intervention participants (4%) were more likely to intend to start chemoprevention than standard control participants (3%), Χ^2^(2) = 6.38, *P *= 0.041 (Table 2).

At post-test, participants in the intervention group were not more likely to demonstrate alignment of attitudes and intentions than those in the standard control group, X^2^(1) = 0.077, *P *= 0.78 (data not shown).

### Post-test informed choice

In the intervention group, 53% of the decisions about intentions to take chemoprevention were informed, in that participants had both sufficient knowledge and alignment of attitudes and intentions (Table 3). In the majority of cases (89%), these were decisions of women who had sufficient knowledge, negative attitudes toward uptake and no intentions to take tamoxifen or raloxifene. Uninformed decisions were mainly due to low levels of decision-relevant knowledge. Compared to intervention participants, fewer standard control participants (6%) made an informed decision (X^2^(1) = 71.97, *P *<0.001, Table 3). Most (93.1%) standard control participants did not have sufficient knowledge to make an informed decision (Table 2).

**Table 3:** Informed decisions: post-test, latent, and three-month follow-up, frequencies (%)

		Intervention (*n *= 383)	Standard control (*n *= 102)	Three-month control (*n *= 100) ^a^	Between-groups differences
**Post-test informed decisions**^b^					

	Negative attitudes, uptake unlikely	180 (47.0%)	4 (3.9%)	-	
	Neutral attitudes, uptake neither likely nor unlikely	15 (3.9%)	2 (2.0%)	-	
	Positive attitudes, uptake likely	7 (1.8%)	0	-	
Total post-test informed decisions		202 (52.7%)	6 (5.9%)		X^2 ^(1) = 71.97, *P *<0.001

**Follow-up informed decisions**^b^					

	Negative attitudes, decided against drug	48 (12.5%)	6 (5.9%)	1 (1.0%)	
	Neutral attitudes, no decision made	17 (4.4%)	6 (5.9%)	7 (7.0%)	
	Positive attitudes, decided to take drug^c^	0	0	0	
Total follow-up informed decisions		65 (16.9%)	12 (11.8%)	8 (8.0%)	X^2 ^(2) = 5.40, *P *= 0.067

**Latent knowledge informed decisions**^b d ^					

	Negative attitudes, decided against drug	131 (34.2%)	3 (2.9%)	-	
	Neutral attitudes, no decision made	36 (9.4%)	0	-	
	Positive attitudes, decided to take drug^c^	0	0	-	
Total latent informed decisions		167 (43.6%)	3 (2.9%)		X^2 ^(1) = 58.54, *P *<0.001

^a ^Three-month control group did not complete measures at post-test; In all cases of informed decision making level of knowledge needs to be sufficient. Attitudes should align with likelihood of taking the drug. Participants in the three-month control group did not answer knowledge questions at post-test; ^c ^At three3 month follow-up, of the two participants who decided to take raloxifene, one had sufficient knowledge but neutral attitudes and one had insufficient knowledge and positive attitudes. ^d ^Latent informed decisions consisted of sufficient post-test knowledge, and aligned attitudes and decisions at follow-up.

### Follow-up knowledge, attitudes and behavior

In the follow-up survey, we assessed knowledge, attitudes and uptake of tamoxifen and raloxifene over the previous three months. While 62% of the intervention group met criteria for sufficient knowledge at post-test, only 25% met these criteria at follow-up, showing a steep decline in retention of information (Table 2). Sufficient knowledge was demonstrated by 15% of standard control participants and 12% of three-month control participants. Differences between the intervention and control groups were statistically significant, (Χ^2 ^(2) = 10.71, *P *= .005).

Attitudes toward uptake of chemoprevention were generally negative in all groups (75% intervention, 70% standard control, 63% three-month control (see Table 2) and did not differ across groups, X^2^(4) = 7.84, *P *= .098.

Intervention participants were more likely to report having made a decision (versus being undecided) about starting chemoprevention, Χ^2^(4) = 29.06, *P *<.001. Among intervention participants, two women (1%) decided to take raloxifene, 45% were undecided, and 55% decided against chemoprevention drugs. No standard control participants decided to take a drug (0%), 66% were undecided and 34% decided against chemoprevention drugs. No three-month control group participants decided to take a drug (0%), 70% were undecided and 30% decided against chemoprevention drugs (Table 2).

Participants in the intervention group most often showed alignment between attitudes and decisions (68% intervention, 55% standard control, 54% three-month control), X^2^(2) = 9.85, *P *= 0.007 (data not shown).

### Follow-up informed choice

At three months, 17% of the intervention group's choices about uptake of chemoprevention could be considered 'informed' according to our definition (Table 3). These were all decisions to not take tamoxifen or raloxifene. Two women decided to use raloxifene, but these choices were uninformed because they either had insufficient knowledge (*n *= 1) or a neutral attitude (*n *= 1). In the control groups, 12% (standard control) and 8% (three-month control) of choices were informed (Table 3). Differences in rates of informed choices between groups were statistically insignificant, Χ^2^(2) = 5.40, *P *= 0.067.

### Latent informed choices

Because knowledge was so low at follow-up and because it is likely that people made decisions based on post-test (latent) knowledge [23] we also examined informed decision making at three months using the post-test levels of sufficient knowledge. This way, 44% of the intervention group's choices about uptake of chemoprevention at three months could be considered 'informed', whereas only 3% of the standard control groups' choices were informed, Χ^2^(1) = 58.54, *P *<0.001 (Table 3).

## Discussion

In this study, Marteau's model [17] was used to determine whether the DA increased the number of informed choices about uptake of tamoxifene and raloxifene. In fact, our data revealed that women given a DA were more likely to have made an informed choice about uptake of tamoxifen and raloxifene than women who did not view the DA. Also, fewer women in the intervention group remained undecided about uptake of the drugs. As expected, three months after baseline the rate of informed decision making about actual uptake was still marginally higher in women who had received the DA, particularly because of declining knowledge levels. However, overall, levels of informed decision making are strikingly low.

Since most respondents had negative attitudes toward uptake of tamoxifen and raloxifene and most decided not to take chemoprevention drugs, uninformed choices were mainly related to insufficient knowledge. One potential reason for relatively low rates of knowledge in the group receiving the DA is that each drug had five risks and two benefits presented. The amount of information conveyed may have been overwhelming and reduced people's recall, even though we asked about gist-level information [23].

The rate of informed choices in the intervention group decreased from 54% (post-test) to 18% (three-month follow-up), but attitudes did not change from post-test to follow-up. The observed decline in informed choices is mainly due to decreased knowledge scores over time and measuring latent decision-making appears to be useful. This raises an interesting question - how long are decision-makers supposed to remember gist-level information once they have come to a decision?

Another issue is the important influence of the definition of what exactly constitutes 'sufficient decision-relevant knowledge' on the resulting proportion of informed choice. Consensus is needed on the levels of knowledge and specific content necessary to make informed decisions. A drawback of the complicated nature of the information being provided, and thus a limitation of this study, is that there were not enough participants with more than 50% knowledge retention to examine differences between groups.

One might expect that women who consider taking chemoprevention drugs are more likely to remember information because it is relevant to their decision, whereas women who have already decided not to take the drugs may be less motivated to remember the information. However, we did not find this effect.

Traditionally two kinds of informed choices are distinguished: sufficient decision-relevant knowledge combined with either positive attitudes and acceptance of the preventive measure or negative attitudes and a decline of the preventive measure [17]. We identified a third type of informed choice, which is the combination of sufficient knowledge, neutral attitudes and being undecided about uptake of the preventive measure. The question is whether this third combination indeed reflects an informed choice rather than ambivalent attitudes, since -- as shown by earlier research -- ambivalence may lead to postponing decision making [26]. We recommend further study into this topic.

It has often been shown that DAs lead to increased levels of knowledge in those who have used them [27]. While many studies of DAs examine changes in knowledge, attitudes, intentions and behavior, few examine the relationship between knowledge and the concordance between attitudes, intentions, and behavior. Even for well-known population-based screening programs, such as breast or cervical cancer screening, interventions specifically aimed at improving informed decision-making are limited [28,29]. This study, therefore, represents a valuable contribution to the literature. Our study has several limitations related to generalizability of the findings. First, despite oversampling eligible African American women, participants were mostly White. Other chemoprevention studies have also encountered this problem [1,5,6,10,21]. Second, we used an online DA, which can ultimately increase intervention reach, but lack of Internet access or discomfort with using the Internet likely narrowed the pool of potential participants and possibly directed access to a more educated sample. However, findings of previous research comparing Internet samples to other samples are inconsistent in this regard [30,31]. Third, we assessed attitudes with one item. Although this single item enabled the registration of negative attitudes considering own participation in the intervention - which is often not the case when attitudes are being assessed - we acknowledge that using one item is a bit limited.

While the above limitations decrease external validity of our study, including two control groups significantly strengthens internal validity. The standard control group allows us to test the effects of the DA compared to not receiving any information about tamoxifen and raloxifene, while the three-month control group allows us a clean control group to evaluate the impact of the DA over time. Our results may generalize to other insured patients, as we recruited participants from two geographically diverse health plan populations.

### Practice implications

Women given a DA describing risks and benefits of tamoxifen and raloxifene were significantly more likely to make informed decisions about undergoing chemoprevention for breast cancer. The intervention materials impacted not only knowledge but also alignment of attitudes with decisions. The online DA format makes this a potentially valuable adjunct in clinical practice, since women could utilize it prior to clinic visits. Finally, this study suggests a valuable way to evaluate the effectiveness of DAs.

## Conclusions

While informed decisions cannot, by definition, be made without sufficient relevant knowledge [17], it is also important that attitudes align with intentions and actual behavior. In this study, women in the intervention group more often had made decisions about uptake of tamoxifen and raloxifene that aligned with their attitudes.

## Abbreviations

BCRAT: Breast Cancer Risk Assessment Tool; DA: decision aid; MMIC: Multidimensional Measure ofInformed Choice; STAR: Study of Tamoxifen and Raloxifene.

## Competing interests

The authors declare that they have no competing interests.

## Authors' contributions

IJK and AFF performed the statistical analysis and drafted the manuscript. PU, BZF and DS conceived of the study and participated in its design and coordination. SMG, JBM and SHA participated in the design and coordination of the study and contributed to the data-collection. AF conceived of the study, participated in its design and coordination and participated in drafting the manuscript. All authors have been involved in revising the manuscript. All authors have read and approved the final manuscript.
